# Addressing Barriers to Care in Odontogenic Infections: The Impact of Timely Surgical Intervention on Reducing Hospital Readmissions in Vulnerable Populations

**DOI:** 10.7759/cureus.86662

**Published:** 2025-06-24

**Authors:** Fawaz Hussain, Aabid Mohiuddin, Alex Huang, Lea Monday

**Affiliations:** 1 Internal Medicine, Detroit Medical Center/Wayne State University, Detroit, USA; 2 Pediatric Infectious Diseases, Detroit Medical Center/Wayne State University, Detroit, USA; 3 Division of Infectious Diseases, Wayne State University School of Medicine, Detroit, USA

**Keywords:** deep neck infection, low socioeconomic status, odontogenic infections, oral maxillofacial, surgical source control

## Abstract

Odontogenic infections are a leading cause of deep neck infections, with their incidence expected to rise due to the prevalence of contributing risk factors, such as uncontrolled diabetes mellitus and obesity. While the existing literature primarily focuses on the management of odontogenic infections in admitted patients, this study uniquely assesses clinical outcomes across all care settings and among a predominantly low socioeconomic status (SES) patient population. These data showed that definitive surgical source control (SC) was associated with an 88% reduction in the relative risk of 30-day all-cause readmission. The data further showed that, in patients admitted due to an odontogenic infection, 87% of those who had SC deferred until outpatient follow-up failed to return. Barriers such as lack of transportation, limited insurance coverage, and poor health literacy can contribute to this low follow-up rate. Therefore, in patient populations with predominantly low SES, clinicians must consider the benefit of immediate SC and the risk of the patient being lost to follow-up.

## Introduction

Odontogenic infections are the leading source of deep neck infections worldwide, often originating from the polymicrobial flora of untreated dental caries or periodontal disease extending into surrounding bone and soft tissue structures [1,2]. Patients typically present with localized erythema, swelling, and pain of the affected tooth, which may advance to abscess formation and local or systemic spread. Patients with a history of poor oral hygiene, uncontrolled diabetes mellitus, immunosuppression, alcohol use, tobacco use, and obesity are at increased risk of developing odontogenic infection [3,4]. In addition, there is an increased epidemiological burden of odontogenic infection among patients with low socioeconomic status (SES), likely related to barriers in accessing routine dental care and increased chronic medical comorbidities [5]. Surgical source control (SC) and antibiotic therapy are fundamental to the effective management and resolution of odontogenic infections. Delayed or inadequate therapy may lead to potentially fatal complications such as cavernous sinus thrombosis, necrotizing fasciitis, Lemierre’s syndrome, and airway obstruction [6]. 

Despite the significant disease burden of odontogenic infection in the low-SES population, there is limited literature examining the impact of SC timing and delivery on clinical outcomes across all care settings among predominantly low-SES patients. This study aims to assess how the timing and setting of surgical SC (inpatient versus outpatient) impact 30-day readmission rates and mortality among all patients presenting with odontogenic infection to a large urban safety-net hospital over a 14-month period. 

## Materials and methods

**Figure 1:**
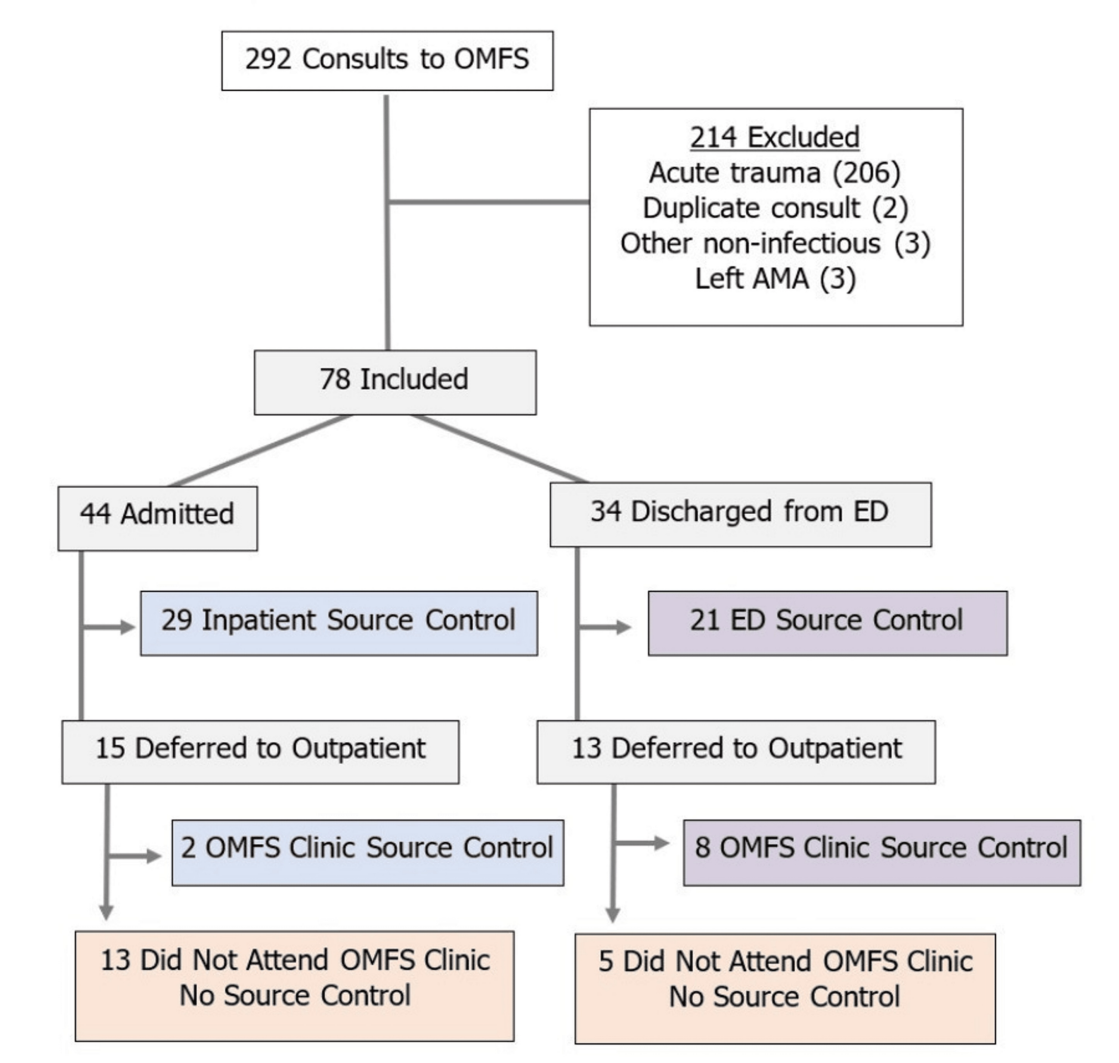
Flowchart of Patients With Odontogenic Infection: Surgical Source Control Across Care Settings Abbreviations: ED, Emergency department; OMFS, Oral and maxillofacial surgery; AMA, Against medical advice

This retrospective observational cohort study analyzed all adult patients evaluated by the Oral and Maxillofacial Surgery (OMFS) team for odontogenic infection at a large urban safety-net hospital in Detroit, MI, within a 14-month period. Odontogenic infection was defined as infection of the jaw, neck, or face originating from a tooth or its supporting structures. Non-infectious consults were excluded (Figure 1). Patients meeting these eligibility criteria were then separated into two cohorts, based on whether they received surgical SC (defined as tooth extraction ± abscess drainage), regardless of whether it was performed in the ED, inpatient, or outpatient setting. Cases were identified through OMFS consult logs, and chart review was performed using a standardized data collection form. Demographic and clinical characteristics retrospectively collected through electronic chart review included age, gender, race, insurance payor status, zip code, medical comorbidities, selected antibiotics, timing of surgical SC, medical disposition, presence of bacteremia, and ICU admission. Primary outcomes measured included the incidences of readmission and mortality within 30 days. 

Continuous variables were reported as median and interquartile range (IQR) and compared using the Wilcoxon rank-sum test for non-parametric data. Categorical data were reported as numbers and percentages, and compared using the chi-square test or Fisher’s exact test for small samples. An acceptable type I error rate for all analyses was α = 0.05. All analyses were performed using Stata software (version 17; StataCorp LLC, College Station, TX, USA). Since the study evaluated practice patterns to identify areas for improvement and cases lost to follow-up, and only collected de-identified, retrospective data, it was classified as a Quality Improvement (QI) project. Therefore, no IRB approval was required. 

## Results

Of the total consults (n = 292) received by the OMFS team during the 14-month study period, 78 adult patients were evaluated for odontogenic infection, meeting the eligibility criteria. There were 214 excluded patients, most often due to the consultation being related to facial trauma without any suspected infection (Figure 1). Included patients had a median age of 39 (IQR 31-55); 52.6% were female, 75.6% were Black patients, and 29.5% lived in low-income zip codes below 125% of the poverty line (Table 1).

**Table 1:** Clinical Characteristics and Outcomes in Odontogenic Infection Patients With and Without Surgical Source Control Wilcoxon rank-sum test for non-parametric continuous variables; chi-square or Fisher’s exact test for categorical variables, as appropriate. Significance level is defined as p < 0.05. *Defined as known human immunodeficiency virus, solid organ or stem cell transplant, lupus, sickle cell disease, or taking chronic steroids; ¥ Included vancomycin, daptomycin, or ceftaroline. Abbreviations: ER, Emergency room; IQR, Interquartile range; MRSA, Methicillin-resistant *Staphylococcus aureus *

Characteristic	Total (n = 78)	Source Control (n = 60)	None (n = 18)	p-value
Age, median (IQR) years	39 (31-55)	39 (31-55)	38 (31-53)	1
Male, n (%)	37 (47.4)	32 (53.3)	5 (27.8)	0.057
Self-Described Race/Ethnicity, n (%)				0.952
Black patients	59 (75.6)	45 (75.0)	14 (77.8)	-
White patients	14 (17.9)	11 (18.3)	3 (16.7)	
Asian patients	1 (1.3)	1 (1.7)	0 (0)	
Unclear patients	4 (5.2)	3 (5.0)	1 (5.5)	
Low-Income Zip Code, n (%)				
≤125% poverty level	23 (29.5)	16 (26.7)	7 (38.9)	0.319
Below state median	67 (85.9)	52 (86.7)	15 (83.3)	0.722
Payor, n (%)				0.301
Medicaid	52 (66.7)	40 (66.7)	12 (66.7)	-
Medicare	14 (17.9)	11 (18.3)	3 (16.7)	
Uninsured	5 (6.4)	4 (6.7)	1 (5.5)	
Commercial	7 (9.0)	5 (8.3)	2 (1.1)	
Comorbidities, n (%)				
Diabetes	9 (11.5)	8 (13.3)	1 (5.6)	0.365
Immunosuppression*	7 (9.0)	4 (6.7)	3 (16.7)	0.193
Empiric Antibiotic Selected, n (%)				
Amp/Sulbactam alone	52 (66.7)	38 (63.3)	14 (77.8)	0.193
Clindamycin alone	6 (7.7)	6 (10.0)	0 (0.0)	0.163
Anti-MRSA coverage^¥^	10 (12.8)	9 (15.0)	1 (5.6)	0.293
Anti-pseudomonas coverage	6 (7.7)	4 (6.7)	2 (11.1)	0.535
Definitive Antibiotic Selected, n (%)				
Amoxicillin/Clavulanate	52 (66.7)	38 (63.3)	14 (77.8)	0.254
Amoxicillin	10 (12.8)	9 (15.0)	1 (5.6)	0.293
Clindamycin	4 (5.1)	4 (6.7)	0 (0)	0.163
Fluoroquinolone	4 (5.1)	3 (5.0)	1 (5.6)	0.667
Other Factors, n (%)				
Infectious diseases consulted	26 (33.3)	21 (35.0)	5 (27.8)	0.569
Blood cultures obtained	36 (46.2)	28 (46.7)	8 (46.2)	0.868
Blood cultures positive	5 (6.4)	4 (6.7)	1 (5.6)	0.866
Required ICU-level care	9 (11.5)	9 (15)	0 (0)	0.081
Disposition, n (%)				0.096
Discharged from ER	34 (43.6)	29 (48.3)	5 (27.8)	-
Admission to hospital	44 (56.4)	31 (51.7)	13 (72.2)	
Source Control Timing, n (%)				
During index discharge from ED	21 (26.9)	21 (35.0)	n/a	n/a
During index admission	29 (37.2)	29 (48.3)	n/a	n/a
Deferred from ED to clinic	8 (10.3)	8 (13.3)	n/a	n/a
Deferred from inpatient to clinic	2 (2.6)	2 (3.3)	n/a	n/a
Outcome, n (%)				
30-day all-cause readmission	7 (9.0)	2 (3.3)	5 (27.8)	0.001
Death within 30 days	1 (1.3)	1 (1.7)	0 (0)	0.581

Among the 78 included patients, 44 were admitted to the hospital, while the remaining 34 were discharged from the emergency department (ED). A total of 60 patients (76.9%) underwent surgical SC (Figure 1). Within the group of admitted patients, 29 (66%) underwent inpatient SC; 15 (34%) did not have procedures, and SC was ultimately deferred to the outpatient setting. Of those 15 patients, only two returned as scheduled to the OMFS clinic for outpatient SC, while the other 13 were lost to follow-up. Within the group of 34 patients discharged from the ED, 21 (62%) underwent surgical SC while in the ED; 13 (38%) were deferred to outpatient SC, of whom eight did return for outpatient SC. 

Patient characteristics and outcomes were compared between the two cohorts - with and without surgical SC - regardless of admission status or SC location, as shown in Table 1. The cohorts did not differ significantly in demographic and clinical characteristics, antibiotic use, or rates of ID consultation. However, there was a trend for SC patients to be male (p = 0.057) and to require ICU-level care (p = 0.081). Notably, there was a statistically significant difference in 30-day readmission rates between patients who underwent SC (3.3%) and those who did not (27.8%) (p = 0.001). This corresponds to an 88% relative risk reduction (95% CI: 31.2% to 97.9%) in 30-day readmission among patients who underwent SC. There was no significant difference in 30-day mortality rates between the two cohorts. These findings highlight the importance of timely surgical SC in reducing 30-day readmission rates among patients with odontogenic infections. 

## Discussion

Despite their high prevalence and potentially catastrophic complications such as endocarditis, odontogenic infections are underrepresented in the infectious diseases literature. SC of any infection is a crucial concept in infectious disease literature, though this term lacks a standardized definition and is not well described outside the realm of sepsis and septic shock [7]. Within the current literature, the majority of studies focus solely on the outcomes of patients with odontogenic infections admitted to the hospital [2,3,8,9]. However, these studies lack generalizability to broader patient populations, including those treated in outpatient and emergency settings. According to a study published in the Journal of Oral and Maxillofacial Surgery, only about one-third of patients presenting to the ED with odontogenic infections are admitted, with insurance status significantly influencing admission rates [10]. The authors noted that many patients undergo quick incision and drainage (I&D) or dental extractions in the emergency room, while others are instructed to follow up in the outpatient setting. While a higher proportion of patients were admitted in our study (44 of 78, or 56%), SC was ultimately delayed in many, with an overall similarly low incidence of inpatient SC of 37%. This study adds to the current literature by describing the clinical outcomes of all patients presenting to an urban health system with odontogenic infection - including demographics and geographic income data - and further determines if those not receiving surgical SC have adverse outcomes. 

This study demonstrated that definitive SC significantly reduced 30-day all-cause readmissions (p = 0.001), with an 88% relative risk reduction of readmission among those who underwent SC. This reduction in 30-day readmission risk among patients with SC may be explained by more complete infection resolution at the initial visit, thus minimizing recurrence or progression of disease. Surgical I&D with focus extraction is the cornerstone of effective therapeutic management of odontogenic infections [11]. Early surgical SC has been shown to minimize the risk of life-threatening complications, shorten the hospital length-of-stay, and reduce overall treatment costs [12]. Notably, patients who had to wait more than 48 hours from presentation to receive surgical SC were at higher risk of requiring re-drainage of the initial infection [13]. This study further reinforces the importance of timely surgical SC to achieve improved therapeutic outcomes, specifically among patients of low SES. 

These findings also emphasized the poor follow-up rates for patients deferred to outpatient surgical SC, particularly among initially admitted patients. Of the 78 total patients in the sample, the infection was severe enough to warrant inpatient admission in 56% of cases (44 patients); 15 of those patients had SC deferred to outpatient care; however, 87% (13 patients) were lost to follow-up. To address this gap in follow-up, clinicians must understand the social determinants of health and the barriers that patients with low SES face in accessing quality healthcare and dental care. These barriers include a lack of transportation, limited insurance coverage, language barriers, and poor health literacy. Patients with poor health literacy are less likely to attend follow-up appointments and adhere to discharge instructions [14,15]. Providing patients deferred for outpatient treatment with comprehensible, multilingual, and culturally sensitive resources that emphasize the importance of surgical SC in the resolution of their infection will help augment their understanding of the treatment plan and likely improve follow-up rates for outpatient SC [16]. Minimizing barriers to accessing preventive dental care services may also help lower the overall burden of odontogenic infections within this population. 

Limitations

This study has a retrospective observational design, which inherently limits the causality between timely surgical SC and reduced hospital readmission. In addition, there may be potential confounding variables that were not adjusted for in the study's analysis, which could have influenced the primary outcome. Lastly, this study was conducted at a single urban safety-net hospital, which reduces the generalizability of these findings to other clinical settings with different patient populations and resources. However, this study still provides meaningful insight into the real-world outcomes of odontogenic infections and highlights the significance of timely SC in reducing readmission risk within a vulnerable urban population.

## Conclusions

There is almost no literature on odontogenic infections in the infectious disease field, despite their common occurrence in the United States due to income inequity and a lack of routine dental coverage. There should not be such a gap in odontogenic infections in the infectious disease literature, particularly regarding the outcomes of how, when, and where these infections are managed. This study uniquely contributes to the understanding of odontogenic infections by evaluating the importance of timely surgical SC on patient outcomes. Given that SC reduces 30-day all-cause readmission rates, minimizes life-threatening complications, and reduces overall treatment costs, clinicians should prioritize timely surgical SC at the initial visit in patients with low SES. Efforts to provide more definitive SC and to address gaps in follow-up are likely to improve individual patient outcomes as well as reduce the healthcare burden in underserved communities. 
